# NPRL2 Inhibition of mTORC1 Controls Sodium Channel Expression and Brain Amino Acid Homeostasis

**DOI:** 10.1523/ENEURO.0317-21.2022

**Published:** 2022-03-02

**Authors:** Jeremy B. Hui, Jose Cesar Hernandez Silva, Mari Carmen Pelaez, Myriam Sévigny, Janani Priya Venkatasubramani, Quentin Plumereau, Mohamed Chahine, Christophe D. Proulx, Chantelle F. Sephton, Paul A. Dutchak

**Affiliations:** Department of Psychiatry and Neuroscience, CERVO Brain Research Centre, Université Laval, Quebec City, Quebec G1J 2G3, Canada

**Keywords:** epilepsy, GATOR1, mTORC1, neurometabolism, NPRL2, sodium channels

## Abstract

Genetic mutations in nitrogen permease regulator-like 2 (NPRL2) are associated with a wide spectrum of familial focal epilepsies, autism, and sudden unexpected death of epileptics (SUDEP), but the mechanisms by which NPRL2 contributes to these effects are not well known. NPRL2 is a requisite subunit of the GAP activity toward Rags 1 (GATOR1) complex, which functions as a negative regulator of mammalian target of rapamycin complex 1 (mTORC1) kinase when intracellular amino acids are low. Here, we show that loss of NPRL2 expression in mouse excitatory glutamatergic neurons causes seizures before death, consistent with SUDEP in humans with epilepsy. Additionally, the absence of NPRL2 expression increases mTORC1-dependent signal transduction and significantly alters amino acid homeostasis in the brain. Loss of NPRL2 reduces dendritic branching and increases the strength of electrically stimulated action potentials (APs) in neurons. The increased AP strength is consistent with elevated expression of epilepsy-linked, voltage-gated sodium channels in the NPRL2-deficient brain. Targeted deletion of NPRL2 in primary neurons increases the expression of sodium channel *Scn1A*, whereas treatment with the pharmacological mTORC1 inhibitor called rapamycin prevents *Scn1A* upregulation. These studies demonstrate a novel role of NPRL2 and mTORC1 signaling in the regulation of sodium channels, which can contribute to seizures and early lethality.

## Significance Statement

Nitrogen permease regulator-like 2 (NPRL2) is a requisite subunit of the epilepsy-linked GAP activity toward Rags 1 (GATOR1) complex that functions as a negative regulator of mammalian target of rapamycin complex 1 (mTORC1) kinase when intracellular amino acids are limited. Here, we report the generation and characterization of a new neurologic model of GATOR1-dependent mTORopathy, caused by the loss of NPRL2 function in glutamatergic neurons. Loss of NPRL2 increases mTORC1 signal transduction, significantly alters amino acid homeostasis in the brain, and causes sudden unexpected death of epileptics (SUDEP). In addition, loss of NPRL2 increases the strength of electrically stimulated action potentials (APs) and the expression of epilepsy-linked sodium channels. These data reveal an unanticipated link between intracellular amino acid signaling by NPRL2 and a novel mTORC1-dependent regulation of sodium channel expression in epilepsy.

## Introduction

Nitrogen permease regulator-like 2 (NPRL2) is a requisite subunit of GAP activity toward Rags 1 (GATOR1), an evolutionarily conserved complex that is comprised of three proteins called NPRL2, NPRL3, and DEP domain containing 5 (DEPDC5). Recent genomic studies have identified mutations in all subunits of GATOR1 that collectively represent ∼10% of the epileptic and autistic population (Dibbens et al., 2013; Ricos et al., 2016; Baldassari et al., 2019). Mutations in the DEPDC5 have also been suggested to be a causal factor for sudden unexpected death of epileptics (SUDEP) in humans (Nascimento et al., 2015). Individuals with GATOR1 mutations can present with focal cortical dysplasia, with defects in cortical lamination and the presence of dysmorphic neurons (Kabat and Król, 2012; Scerri et al., 2015; Weckhuysen et al., 2016; Chen et al., 2017). These malformations have been found to occur through a two-hit genetic mechanism (Ribierre et al., 2018), confounding the specific GATOR1-dependent mechanisms that contribute to neurologic dysfunction.

GATOR1 functions as a negative regulator of mammalian target of rapamycin complex 1 (mTORC1), a dynamic protein kinase that controls cellular growth, protein translation, and metabolic processes in cells (Bar-Peled et al., 2013; Liu and Sabatini, 2020). mTORC1 activity is controlled by distinct upstream regulatory protein complexes comprised of GATOR1 and TSC1/2, which function as GTPase activating proteins toward small GTP-binding proteins on the lysosomal surface called RAGs and Rheb, respectively (Meikle et al., 2007; Sancak et al., 2010; Efeyan et al., 2013). The interaction of mTORC1 with GTP-bound RAG and Rheb contributes to the activation of its kinase function (Laplante and Sabatini, 2009). Mutations in either GATOR1 or TSC1/2 are generally classified as mTORopathies, which present with overlapping neurologic hallmarks, including seizures and autism spectrum disorders (Talos et al., 2012; Griffith and Wong, 2018). These similarities suggest an important role for strict mTORC1 regulation in the control of brain function (Curatolo et al., 2015; Specchio et al., 2020).

When intracellular amino acids are limited, GATOR1 represses mTORC1-dependent signaling and reduces the anabolic consumption of amino acids (Bar-Peled et al., 2013; Bourdeau Julien et al., 2018; Shen et al., 2019). Previous loss-of-function studies have shown that NPRL2 is necessary to maintain intracellular amino acids homeostasis by regulating the expression of genes important for metabolism of amino acids, including glutamine and glutamate (Dutchak et al., 2015, 2018; Chen et al., 2017). While intracellular glutamate functions as a building block for general protein synthesis, it can also contribute to cellular energy metabolism through mitochondrial anapleurosis or function as an excitatory neurotransmitter of glutamatergic neurons (Zhou and Danbolt, 2014).

Within the brain, glutamine contributes to the synthesis of both excitatory and inhibitory neurotransmitters in glutamatergic or GABAergic neurons, respectively. The metabolism of these neurotransmitters is highly inter-dependent within distinct cell populations, comprising the glutamine-glutamate/GABA cycle to adequately supply neurotransmitters within the central nervous system. This cycle describes the shuttling of glutamine from astrocytes to glutamatergic or GABAergic neurons for neurotransmission, and subsequent re-uptake in astrocytes for glutamine regeneration (Schousboe et al., 2013). Alterations in this cycle are frequently observed in epileptic patients and have been associated with defects in astrocyte glutamine synthesis, or changes in glutaminase and glutamate dehydrogenase (Eid et al., 2016). These metabolic links to epilepsy prompted us to investigate the role of NPRL2 in glutamatergic neurons, which use glutamate as an excitatory neurotransmitter. While the contribution of other GATOR1 proteins, namely DEPDC5, have been explored in the GABAergic neurons in zebrafish (Swaminathan et al., 2018), the role of NPRL2 in glutamatergic neurons and its contribution to the mammalian brain has not been investigated.

Here, we report the generation and characterization of a new neurologic model of GATOR1-dependent mTORopathy, caused by the loss of NPRL2 function in glutamatergic neurons. Loss of NPRL2 is sufficient to increase mTORC1 signaling despite an intact TSC1/2 complex, significantly alter amino acid homeostasis in the brain and cause seizures before early death. In addition, loss of NPRL2 increases the strength of electrically stimulated action potentials (APs) and the expression of epilepsy-linked sodium channels (Lakhan et al., 2009; Kaplan et al., 2016). These data reveal an unanticipated link between intracellular amino acid signaling through GATOR1 and a novel mTORC1-dependent regulation of sodium channel expression.

## Materials and Methods

### Animal experiments

NPRL2 nKO mice were generated by crossing *Nprl2^loxP/+^* mice previously described (Dutchak et al., 2015), with *VgluT2-ires-*Cre females mice (Vong et al., 2011) to generate glutamatergic neuron-specific heterozygous animals. The expression of Cre-recombinase in these mice is regulated by an internal ribosomal entry site that was created using a knock-in strategy targeting the *VgluT2* gene. Heterozygous animals were backcrossed to C57/B6 for at least three generations, with subsequent crosses with *Nprl2^loxP/+^* and *Nprl2^loxP/loxP^* animals, resulting in control mice (*Nprl2^loxP/+^* and *Nprl2^loxP/loxP^*), NPRL2 nHET mice (*Nprl2^loxP/+^*; *VgluT2-ires-*Cre), and NPRL2 nKO mice (*Nprl2^loxP/loxP^*; *VgluT2-ires-*Cre) that were used for these studies. Genotypes of all animals were determined by PCR, using the following primers: *Nprl2* (forward) 5′-CTCAGGTTCTACGCAGTGACTTC-3′, *Nprl2* (reverse) 5′-CATGGCGCTGTCTGGATCC-3′, *Nprl2* (knock-out) 5′-CAGGCTTCATACTTCTACCCTC-3′, *VgluT2* (forward) 5′-AAGAAGGTGCGCAAGACG-3′, *VgluT2* (Cre-reverse) 5′-ACACCGGCCTTATTCCAAG-3′, *VgluT2* (wt-reverse) 5′-CTGCCACAGATTGCACTTGA-3′. Video recordings were captured using a Sony FDR-AX53 camcorder, with the mother present in the cage. All animal experiments were approved by the Université Laval Committee on Ethics and Animal Research.

### Immunohistochemistry

Tissue sections were prepared from P16 animals that were perfused with 4% (wt/vol) formaldehyde and immersion fixed in 4% formaldehyde for 16 h. Floating sections were immunostained with primary antibodies overnight at 4°C using a 1:500 antibody dilution shown in Table 1. Alexa Fluor-conjugated secondary antibodies (Invitrogen) were incubated for 2 h at room temperature before washing and mounting. Three animals per each genotype were analyzed using confocal microscope.

**Table 1:** List of antibodies used in this study

Protein target	Vendor	Catalog #
AKT	Cell Signaling Technology	9272
DEPDC5	Santa Cruz Biotechnology, Inc.	sc-86115
GAPDH	Sigma	G9295
GFAP	Millipore	MAB5541
mGluR1	Fisher Scientific	12370
NeuN	Millipore	AB5541
NPRL2	Santa Cruz Biotechnology, Inc.	sc-376986
NPRL3	Abcam	ab121346
p70 S6 Kinase	Cell Signaling Technology	9202
Phospho-p70 S6 kinase (T389)	Cell Signaling Technology	9205
Phospho-AKT (Ser473)	Cell Signaling Technology	9271
Phospho-rpS6 (Ser240/244)	Cell Signaling Technology	2215
rpS6	Cell Signaling Technology	2217
Scn1A	Cell Signaling Technology	18339
VGLUT2	Synaptic Systems	135404

### Western blot analysis

Tissues were homogenized in lysis buffer containing 150 mm sodium chloride, 50 mm sodium fluoride, 100 μm sodium orthovanadate (pH 10.0), 50 mm sodium pyrophosphate tetrabasic, 10 mm β-glycerophosphate, 5 mm EDTA, 5 mm EGTA, and 10 mm HEPES (pH 7.4) and 0.5% Triton X-100 supplemented with complete anti-protease cocktail (Roche). Lysates were pelleted by centrifugation. Cleared lysates, representing the soluble fraction, and the insoluble membrane fractions were boiled in 1× Laemmli sample buffer and separated on SDS-PAGE for Western blotting using antibodies listed in Table 1.

### Golgi staining

NPRL2 nKO and littermate controls were used for Golgi staining, using the manufacturer’s protocol of the FD Rapid GolgiStain kit (FD Neurotechnologies). 10 cortical neurons (Layers IV–V) from three independent animals per genotype were traced and analyzed using Neurolucida 360 (MBF Biosciences). Sholl analysis was performed starting at 10 μm from the soma with 5-μm intervals as previously described (Sephton et al., 2014).

### Slice preparation for electrophysiology

Mice were first anesthetized with isoflurane. The brain was then quickly dissected and placed in the cutting chamber filled with an ice-cold NMDG-artificial CSF (aCSF) solution containing the following: 1.25 mm NaH_2_PO_4_, 2.5 mm KCl, 10 mm MgCl_2_, 20 mm HEPES, 0.5 mm CaCl_2_, 24 mm NaHCO_3_, 8 mm D-glucose, 5 mm L-ascorbate, 3 mm Na-pyruvate, 2 mm thiourea, and 93 mm NMDG (osmolarity adjusted with sucrose to 300–310 mOsm/l); pH adjusted to 7.4 with HCl 10N. Coronal slices (250 μm) were then cut with a vibratome (VT2000; Leica) to obtain complete sections containing lateral habenula (LHb). Slices were placed in a 32°C oxygenated NMDG-aCSF solution for 10 min before incubation for 1 h at room temperature in HEPES-aCSF solution: 1.25 mm NaH_2_PO_4_, 2.5 mm KCl, 10 mm MgCl_2_, 20 mm HEPES, 0.5 mm CaCl_2_, 24 mm NaHCO_3_, 2.5 mm D-glucose, 5 mm L-ascorbate, 1 mm Na-pyruvate, 2 mm thiourea, 92 mm NaCl, and 20 mm sucrose (osmolarity adjusted to 300–310 mOsm/l at pH 7.4) and finally transferred into a recording chamber on the stage of an upright microscope (Zeiss) where it was perfused at a rate of 3–4 ml/min with aCSF: 120 mm NaCl, 5 mm HEPES, 2.5 mm KCl, 1.2 mm NaH_2_P0_4_, 2 mm MgCl_2_, 2 mm CaCl_2_, 2.5 mm glucose, 24 mm NaHCO_3_, and 7.5 mm sucrose. The aCSF in the perfusion chamber was kept at 32°C. All solutions were aerated with 95% O_2_ and 5% CO_2_ (Ting et al., 2018).

### Whole-cell patch clamp recordings

A water immersion 60× objective and a video camera (Zeiss) were used to visualize neurons in LHb. Whole-cell patch clamp recordings were performed under current clamp with an Axopatch 200B amplifier (Molecular Devices) using borosilicate patch electrodes (3- to 7-MΩ resistance). Pipettes were filled with an intracellular patch solution containing the following: 130 mmol/l K-gluconate, 5 mmol/l KCl, 10 mmol/l HEPES, 2.5 mmol/l MgCl_2_, 4 mmol/l Na_2_-ATP, 0.4 mmol/l Na_3_-GTP, 10 mmol/l Na-phosphocreatine, 0.6 mmol/l EGTA, and 0.2% biocytin (pH 7.35). Signals were filtered at 5 kHz using a Digidata 1500A data acquisition interface (Molecular Devices) and acquired using pClamp 10.6 software (Molecular Devices). Pipette and cell capacitances were fully compensated. The pH and osmolarity were adjusted to 7.3 and 285–290 mOsm/l, respectively. Data were analyzed offline using clampfit 10.6 software. Neurons were selected randomly from the LHb of prepared slices; 1–2 min after obtaining whole-cell configuration, the resting membrane potential (RMP) was recorded in current clamp mode right after whole-cell configuration had been obtained. Rheobase current was assessed by applying depolarizing current steps (from −20 to 100 pA, 5-pA increments and 1-s duration), and it was calculated as the minimum current needed to elicit the AP. To examine evoked firing properties, depolarizing current steps (−20 to +100 pA, 20-pA increments and 300-ms duration) were applied to the cells. APs generated during this period were counted, and we obtained the number of spikes and frequency of firing.

### Metabolite analysis

The cortex of NPRL2 nKO and littermate controls were harvested from living animals at postnatal day 16 (P16) showing no physical signs of stress, by cervical dislocation, rapid dissection, and snap freezing in liquid nitrogen. Samples were stored at −80°C until processing. One hemisphere was subjected to metabolite extraction using the Absolute*IDQ* p180 kit (Biocrates Life Sciences), using the manufacturer’s protocol. Samples were analyzed on a SCIEX 5500 QTRAP by The Analytical Facility for Bioactive Molecules, The Hospital for Sick Children, Toronto, Canada.

### Primary neuron cultures

Primary cortical neurons were prepared from P0-P1 *Nprl2^fl/fl^* mice using previously described methods (Nault and De Koninck, 2010). Dissociated neurons were grown in neurobasal medium supplemented with B27 (Thermofisher), L-Glutamine (Fisher Scientific) and penicillin/streptomycin (Thermofisher). On day in vitro (DIV) 4 cultures were treated with 5 μm AraC (Sigma) and either control or CRE-expressing lentivirus. On DIV7 cells were treated with 10 nm rapamycin or DMSO (vehicle) for 24 h and harvested in TRIzol (Thermofisher) for RNA isolation.

### Quantitative (q)RT-PCR analysis

Total RNA from tissues or cells was extracted using TRIzol (Invitrogen), following the manufacturers protocols. Primer were selected to span exon-exon junctions where possible. RNA extracts were treated with DNase (Roche) and the High Cap cDNA Reverse Transcription kit (Applied Biosystems) was used for cDNA synthesis. qRT-PCR reactions contained 25 ng cDNA, 150 nm each primer pair, and 5 μl SYBR GreenER (Invitrogen). All reactions were performed in triplicate on the QuantStudio 5 Real-Time PCR system (Applied Biosystems). Relative mRNA levels were calculated using the comparative threshold cycle method using U36B4 as the internal control. qRT-PCR primer sequences used in this study are listed in Table 2.

**Table 2:** List of all qRT-PCR primers used in this study

Gene	Forward	Reverse
*Bax*	GTCTCCGGCGAATTGGA	TCCACGTCAGCAATCATCCT
*Dapk2*	GCAGGGAGCAGAGCAGAG	CCTGGACCATACAATCGGCG
*Dapk3*	CGTAGATGCCGTCTCAGGAT	TGGGGACACAGGAGCGTC
*Kv1.1*	CCCGCCAGCGTCCATC	CTGCAGCCCTTCTAGGACAC
*Kv1.3*	CTTCTGGTGCGGTTCTTTGC	TACCTTGTCGTTCAGCCAGC
*Kv1.4*	GGCTTAACAAGTGATCGCTGC	CCAAGGTAGACCCCGGAGAT
*Kv1.6*	GACTGCCAATTTCTGCTTGGG	AGTTATTCAGTGGGGAGGCG
*Kv2.2*	AACGTAGGGGGCCTTAACCA	TTGTAGTCATCGCACACCTCC
*Pcna*	CGAAGGCTTCGACACATACC	GGACATGCTGGTGAGGTTCA
*Scn1A*	CTGGCTGGACTTCACTGTCAT	GATGGTCTTCAGGCCTGGAA
*Scn1B*	AACACCAGCGTCGTCAAGAA	CCATCTCTGCCACAAGCCAT
*Scn2A*	ACCTTTGCGTATGTAACAGAATTT	ACAGCTTCTTCACCGACTGG
*Scn2B*	CGCCTAACGTCACAGTCTCT	GGACACTAAGAGTGGTGGGC
*Scn3A*	GGACGTGGGGTCTGAGAATG	GTGGTACCGTTACTGTTGCG
*Scn3B*	GCTCAGGAAAATGCGTCTGACTAC	TGCCTGTACATCACCTCAAGT
*Scn4B*	GTGGACAACACGGTGACTCT	CACTCCTTCTTCTTCTCTCGGG
*Scn5A*	AAGCTAGGCAATTTGTCGGC	GTCTTCAGGCCTGGAATAACTG
*U36B4*	CGTCCTCGTTGGAGTGACA	CGGTGCGTCAGGGATTG

### Statistical analysis

Statistical analysis was performed by two-tailed Student’s *t* test using Microsoft Excel 2016. A *p*-value of <0.05 was considered significant.

## Results

### Loss of NPRL2 in glutamatergic neurons causes seizures and early lethality

**Figure 1.**
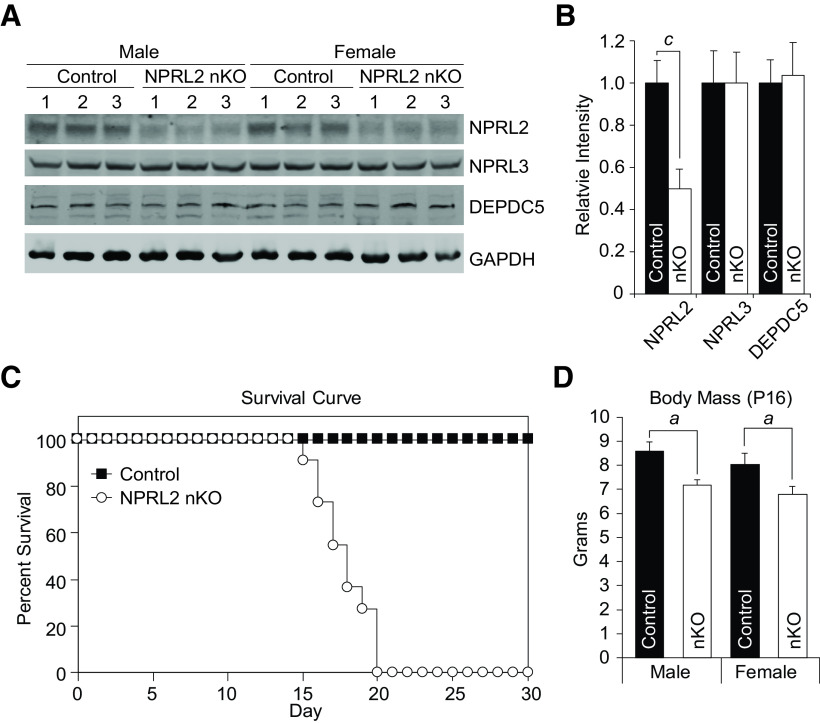
Loss of NPRL2 expression in glutamatergic neurons causes early lethality. ***A***, Western blot analysis of NPRL2, NPRL3, DEPDC5, and glyceraldehyde 3-phosphate dehydrogenase (GAPDH) from brain protein extracts (*n* = 3 per group). ***B***, Quantification of Western blot signal intensities relative to GAPDH (*n* = 6 per genotype). Error bars represent the mean ± SD. ***C***, Survival curve of NPRL2 nKO and wild-type littermate controls (*n* = 12/40, respectively). All NPRL2 nKO animals die 20 d after birth. ***D***, Body mass of NPRL2 nKO and littermate controls (male *n* = 7/16 and female *n* = 7/6, respectively). (Extended Data Fig. 1-1). Error bars represent the mean ± SEM *a*, *p* < 0.05; *c*, *p* < 0.001.

To determine the function of NPRL2 in glutamatergic neurons, we crossed our genetically engineered *Nprl2*-floxed mice with *VgluT2-ires-*Cre females mice to generate *Nprl2^loxP/loxP^; VgluT2-ires-*Cre*^+/WT^* (NPRL2 nKO) mice (Vong et al., 2011; Dutchak et al., 2015). We selected this Cre-driver because it is widely expressed early in developing glutamatergic neurons, permitting us to investigate early onset epileptic phenotypes. To verify NPRL2 deletion, we performed Western blot analysis targeting NPRL2 and other protein subunits of GATOR1, NPRL3, and DEPDC5, in cerebral protein extracts from WT and NPRL2 nKO animals. NPRL2 expression was reduced by 50% in the NPRL2 nKO compared with control, indicating a targeted knock-out of NPRL2 in the VGLUT2 neuron population (Fig. 1*A*,*B*). Consistent with previous observations, loss of NPRL2 did not alter NPRL3 or DEPDC5 expression in the NPRL2 nKO (Dutchak et al., 2015). Animals were born at the expected Mendelian ratios; however, NPRL2 nKO mice did not live beyond 20 d after birth (Fig. 1*C*). We measured the body mass of NPRL2 nKO mice at P16 and found a small but significant reduction in both males and females compared with littermate controls (Fig. 1*D*). To investigate the cause of death, we performed 24-h video recordings of control and NPRL2 nKO littermates, with their mother present. Control mice were unremarkable, whereas all NPRL2 nKO mice began a series of seizures ∼3 h before the start of the 12-h light cycle on the day of their death. Over the next 2–4 h, both tonic and tonic-clonic seizures were observed, lasting 1.6 ± 0.6 and 5.6 ± 1.4 min, respectively (Extended Data Fig. 1-1). NPRL2 nKO pups died following a tonic-clonic seizure lasting >5 min. These observations show that NPRL2 function in glutamatergic neurons is essential for viability, despite the presence of an intact TSC1/2 complex.

10.1523/ENEURO.0317-21.2022.f1-1 Extended Data Figure 1-1 Seizure analysis of NPRL2 nKO mice. Quantification of the tonic and tonic-clonic seizures of the NPRL2 nKO mice before their death. Download Figure 1-1, EPS file.

### NPRL2 contributes to dendritic branching and soma size of neurons

**Figure 2.**
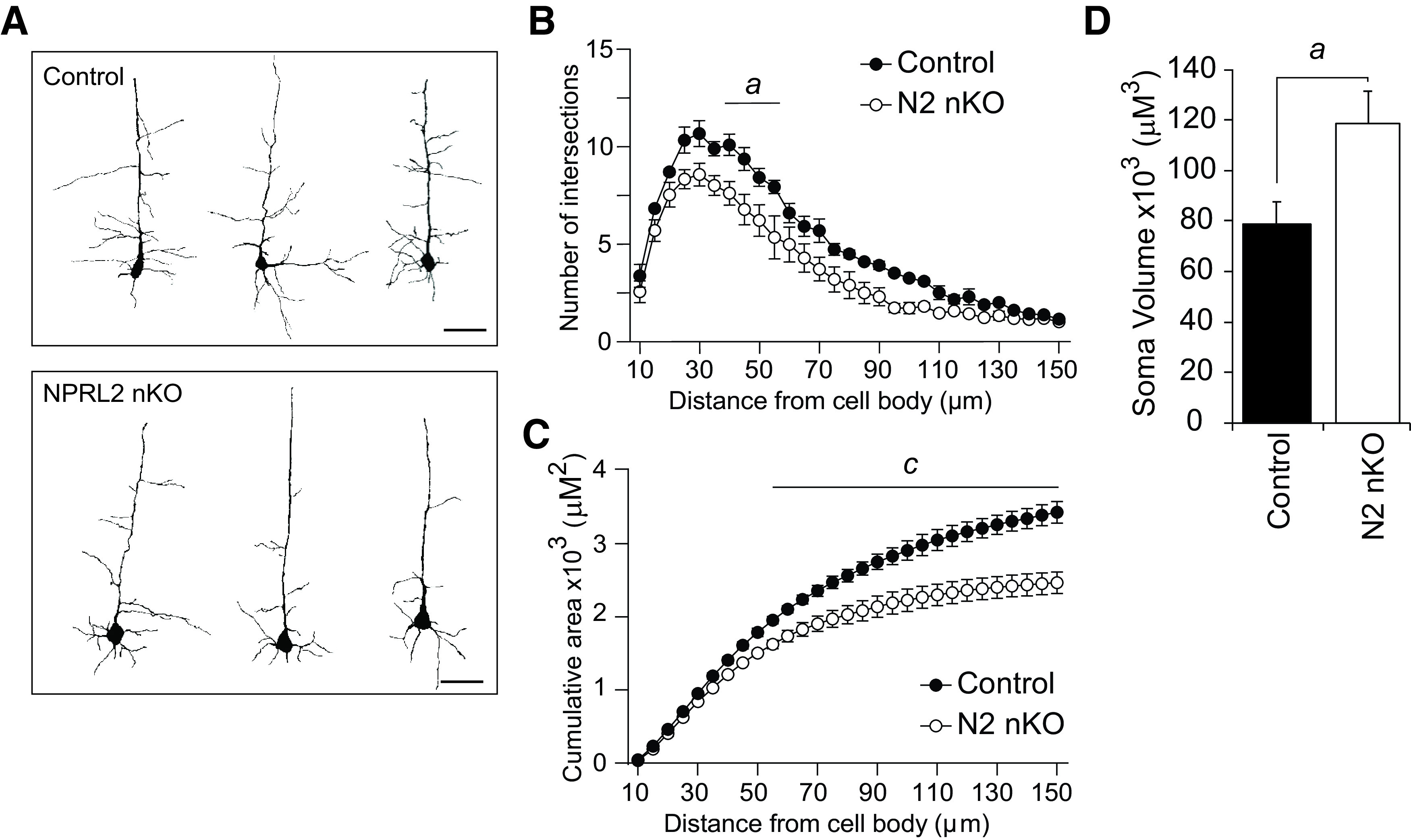
NPRL2 regulates dendritic branching of cortical neurons. ***A***, Representative images of Neurolucida tracing of the dendrites of Layer IV–V cortical neurons in NPRL2 nKO and littermate controls (*n* = 30 neurons/3 mice per genotype). ***B***, Sholl analysis shows a reduced number of intersections, and ***C*** reduced cumulative area of dendrites in the NPRL2 nKO compared with littermate controls at P16. ***D***, Soma volume of NPRL2 nKO versus littermate controls. Error bars represent the mean +/- SEM. Scale bar: 50 μm. *a*, *p* < 0.05; *c*, *p* < 0.001.

Previous studies have shown that deletion of the GATOR1 subunit called DEPDC5, using a pan-neuronal Cre driver, reduces dendritic branching of neurons (Yuskaitis et al., 2018). To determine whether the loss of NPRL2 alters dendritic branching in our model, we performed Golgi staining and Sholl analysis of Layer IV–V cortical neurons in the NPRL2 nKO and littermate controls at P16 (Fig. 2*A*). We observed a significant decrease in the number of intersections and cumulative area of NPRL2 nKO neurons, compared with control (Fig. 2*B*,*C*). We also performed 3D reconstruction of the cell bodies to determine whether the loss of NPRL2 increased the soma size, consistent with established pro-growth effects of mTORC1. We observed the soma of NPRL2 nKO neurons were 1.5-fold larger compared with control (Fig. 2*D*). No gross morphologic differences were observed in the NPRL2 nKO brain compared with controls at P16 (data not shown). These data support NPRL2 as a critical regulator of dendritic branching and neuron size *in vivo.*

### Loss of NPRL2 alters intrinsic electrophysiological properties in neurons

**Figure 3.**
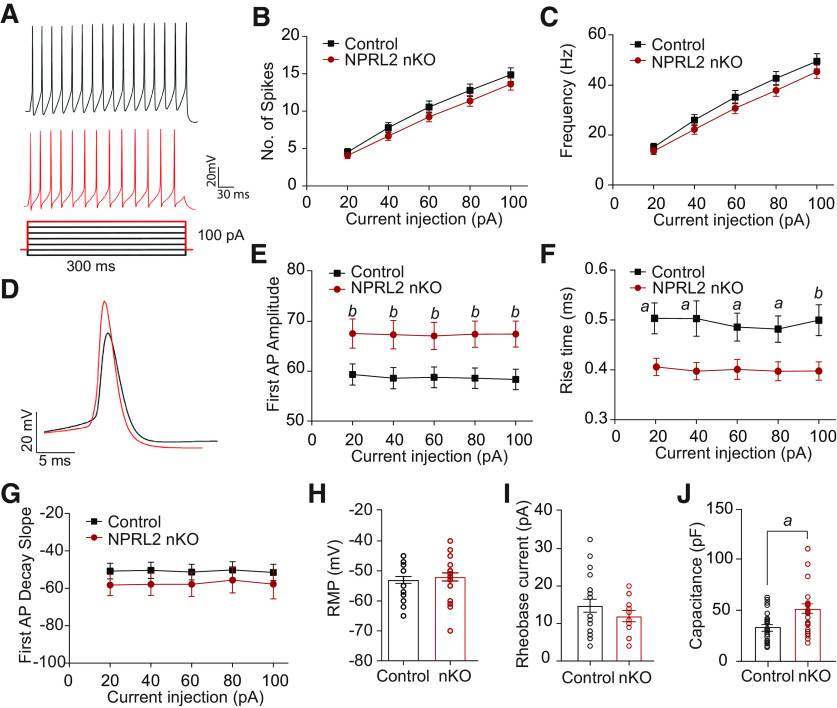
Electrophysiological properties of NPRL2 nKO neurons. ***A***, Representative tracings of the response samples of control (black) or NPRL2 nKO (red) neurons and protocol of injected depolarization steps. ***B***, The number of APs elicited by different current steps and their (***C***) frequency. ***D***, Example trace of the first AP from control (black) or NPRL2 nKO (red). First AP (***E***) amplitude, (***F***) rise time, and (***G***) decay slope evoked by different depolarizing steps. RMP (***H***), rheobase current (***I***), and capacitance (***J***) measurements from NPRL2 nKO and controls. NPRL2 nKO = 23 neurons/3 mice; control = 25 neurons/3 mice. Error bars represent the mean +/- SEM. *a*, *p* < 0.05; *b*, *p* < 0.01.

Mutations in NPRL2 are associated with familial forms of epilepsy (Ricos et al., 2016; Weckhuysen et al., 2016; Baldassari et al., 2019). To determine whether the loss of NPRL2 alters the intrinsic passive and active neuronal electrophysiological properties, we prepared acute brain slices encompassing the LHb, which contain exclusively VGLUT2-positive neurons, from NPRL2 nKO and control littermates. To determine neuronal firing properties, evoked firing activity was evaluated by injecting depolarizing current steps. We observed no significant difference in the number of APs (Fig. 3*A*,*B*), or instantaneous firing frequency (Fig. 3*C*) elicited by progressive depolarizing current steps. However, the electrophysiological properties of the first AP evoked by each depolarizing step showed significantly greater AP amplitude (Fig. 3*D*,*E*), and reduced rise time (Fig. 3*F*) in the NPRL2 nKO neurons compared with littermate controls. No significant difference was observed in the first AP decay slope, RMP, or rheobase current (Fig. 3*G–I*). Consistent with the larger size of the NPRL2 nKO neurons (Fig. 2*D*), a significant increase in capacitance was observed in these cells compared with control (Fig. 3*J*). These data suggest that loss of NPRL2 increases the sodium-dependent depolarization step of the AP.

### NPRL2 controls mTORC1 signal transduction and neurometabolic homeostasis

**Figure 4.**
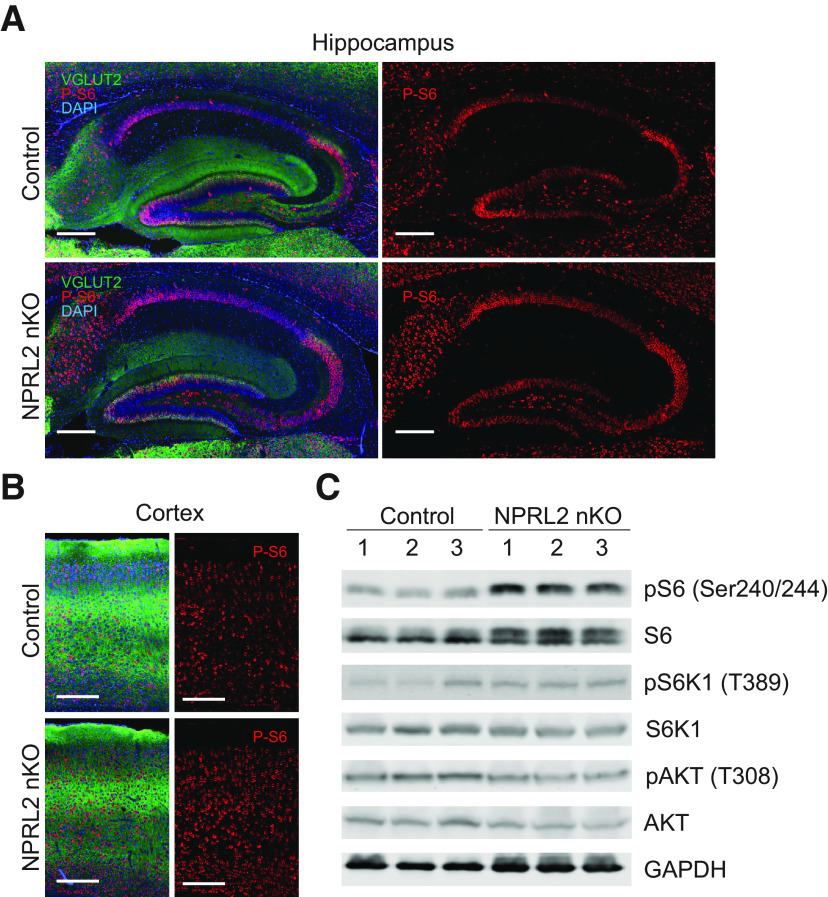
Loss of NPRL2 increases mTORC1 signaling. Representative image of immunohistochemical staining of P-S6 (Ser240/244) and VGLUT2 in the (***A***) hippocampus and (***B***) cortex of NPRL2 nKO and control at P16. ***C***, Western blot analysis of pS6, S6, pS6K1, S6K1, pAKT, AKT, and GAPDH from soluble protein extracts (*n* = 3/3; Extended Data Figs. 4-1, 4-2).

**Figure 5.**
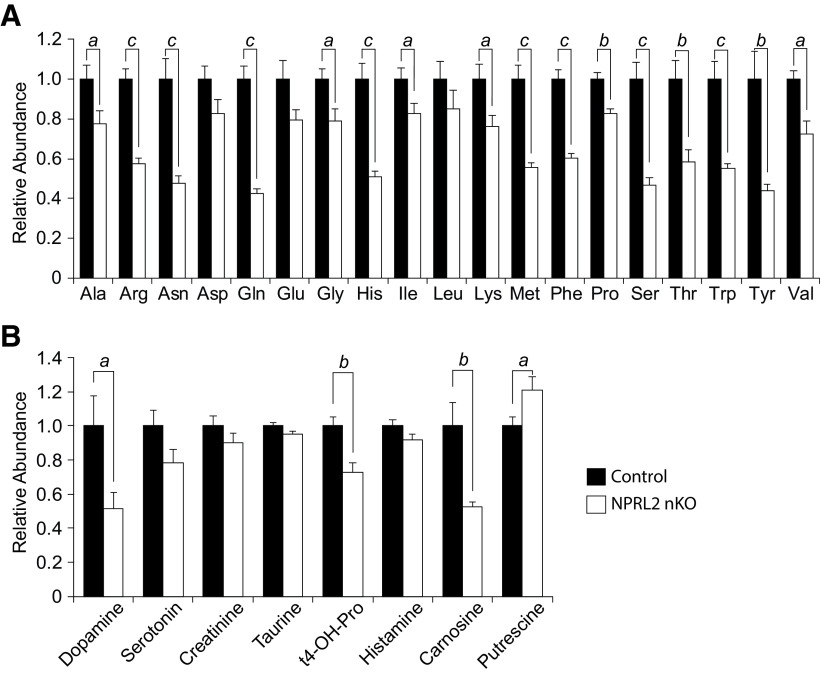
Metabolite analysis of P16 brain extracts. Relative abundance of (***A***) amino acids, and (***B***) biogenic amines were measured from NPRL2 nKO (white bars) and littermate (black bars) controls using LC-MS/MS (*n* = 7/10, respectively). Error bars represent the mean ± SEM (Extended Data Figs. 5-1, 5-2) *a*, *p* < 0.05; *b*, *p* < 0.01; *c*, *p* < 0.001.

NPRL2 is an essential protein subunit of the GATOR1 complex, which functions as a negative regulator of mTORC1 when intracellular amino acids are limited. To determine whether NPRL2 is necessary to repress mTORC1 activity our model, we performed immunohistochemistry and confocal microscopy on sections from NPRL2 nKO and control animals at P16, stained with phosphorylated ribosomal protein S6 (P-S6), a downstream marker of mTORC1 activity, and VGLUT2 (Fig. 4*A*,*B*; Extended Data Fig. 4-1). Increased levels of P-S6 in VGLUT2-positive brain regions were observed in NPRL2 nKO compared with control, suggesting a failure to repress mTORC1 signaling. We also co-stained sections with NeuN, GFAP, and P-S6 to determine whether the effect on P-S6 was cell autonomous. We observed no increase in P-S6 staining in cells co-stained with GFAP in the NPRL2 nKO compared with control, consistent with a cell autonomous effect (Extended Data Fig. 4-2). Western blot analysis of protein extracts showed a significant increase in P-S6 abundance in the NPRL2 nKO compared with WT (Fig. 4*C*; Extended Data Fig. 4-1), consistent with histologic observations. We also observed a trend of increased phosphorylation of S6-kinase, and a small reduction in total AKT and phosphorylated AKT (S473) in the NPRL2 nKO. These data suggest that the regulation of mTORC1 through NPRL2 is necessary to prevent hyperactive mTORC1 signaling in glutamatergic neurons despite an intact TSC1/2 complex. mTORC1 activity is implicated in diverse cellular processes that impact the regulation of metabolic homeostasis within cells. To determine whether the loss of NPRL2 in glutamatergic neurons contributes to metabolic defects in the brain, metabolites were extracted and quantified using targeted small molecule mass spectrometry. NPRL2 nKO extracts showed a significant reduction in the abundance of all amino acids except glutamate, aspartate and leucine, compared with littermate controls (Fig. 5*A*). Remarkably, the abundance of glutamine was 60% lower in the NPRL2 nKO brain extracts, compared with littermate controls. We also observed a significant decrease in dopamine, but no change in serotonin, histamine, creatinine or taurine (Fig. 5*B*). The abundance of carnosine was significantly reduced, while putrescine was significantly increased in the NPRL2 nKO brain (Fig. 5*B*). Since increased levels of putrescine are associate with cell death, we measured the expression of genes that are transcriptionally induced by neuronal cell death, including *Bax*, *Dapk2*, *Dapk3*, and *Pcna*, but found no significant change at P16 (Extended Data Fig. 5-1; Chiang et al., 2001; Takao et al., 2006). No significant differences were observed in the abundance of measured acylcarnitines, sphingolipids, lysophosphatidylcholines (Extended Data Fig. 5-2), nor phosphatidylcholines between NPRL2 nKO and control extracts. Collectively, these data show an important function of NPRL2 in regulating amino acid homeostasis in brain.

10.1523/ENEURO.0317-21.2022.f4-1 Extended Data Figure 4-1 Quantification of (***A***) P-S6 signal intensity from cortical immunohistochemistry staining and (***B***) relative signaling intensity of P-S6:S6 of Western blot analysis. Download Figure 4-1, EPS file.

10.1523/ENEURO.0317-21.2022.f4-2 Extended Data Figure 4-2 Representative image of immunohistochemical staining of P-S6, NeuN, and GFAP in the cortex of (***A***) control or (***B***) NPRL2 nKO at P16 (*n* = 3 per genotype). Scale bar: 25 μm. Download Figure 4-2, EPS file.

10.1523/ENEURO.0317-21.2022.f5-1 Extended Data Figure 5-1 Quantitative gene expression of *Bax*, *Dapk2*, *Dapk3*, and *Pcna,* in control of NPRL2 nKO brain (*n* = 5/4 per genotype, respectively). Download Figure 5-1, EPS file.

10.1523/ENEURO.0317-21.2022.f5-2 Extended Data Figure 5-2 Relative abundance of (***A***) acylcarnitines, (***B***) sphingolipids, and (***C***) lysophosphatidylcholines (*n* = 7/10, respectively). Error bars represent the mean ± SEM. Download Figure 5-2, EPS file.

### NPRL2-dependent mTORC1 activity controls sodium channel expression

**Figure 6.**
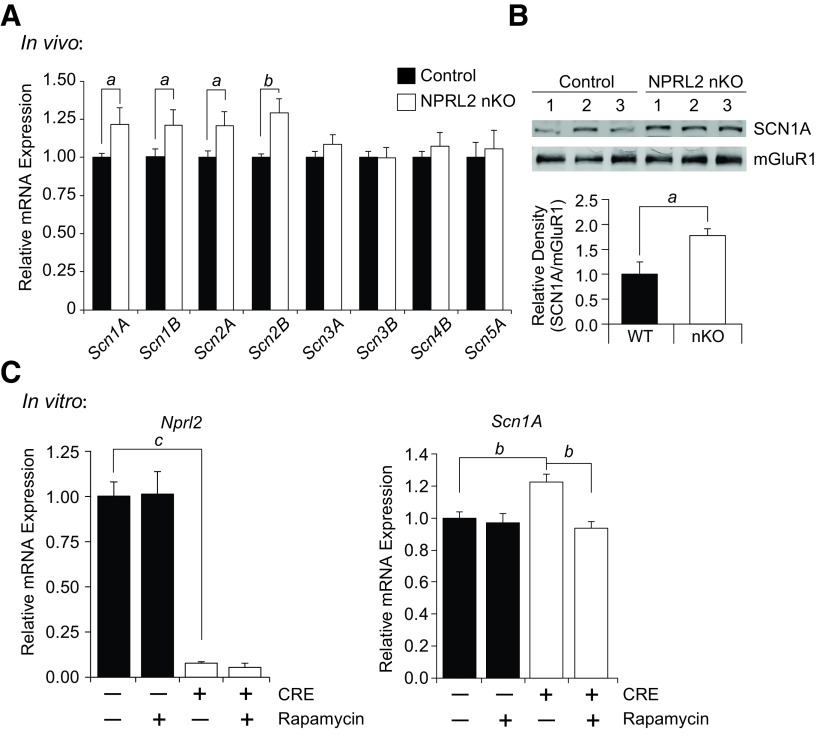
NPRL2 contributes to the regulation of sodium channel expression. ***A***, qRT-PCR analysis of sodium voltage-gated channel α subunits (*Scn1A*, *Scn2A*, *Scn3A*, and *Scn5A*) and sodium voltage-gated channel β subunits (*Scn1B*, *Scn2B*, *Scn3B*, and *Scn4B*) in control and NPRL2 nKO brain at P16 (*n* = 5/4, respectively). ***B***, Western blot analysis of SCN1A and metabotropic glutamate receptor 1 (mGluR1) from membrane fractions of P16 WT and NPRL2 nKO brain (*n* = 3/3). ***C***, qRT-PCR analysis of *Nprl2* and *Scn1A* from *Nprl2^loxP/loxP^* primary neurons infected with control or CRE-expressing lentivirus and treated with 10 nm rapamycin, or DMSO control, for 24 h (*n* = 6/6; 2 independent experiments; Extended Data Fig. 6-1). Error bars represent the mean +/- SEM. *a*, *p* < 0.05; *b*, *p* < 0.01; *c*, *p* < 0.001.

The electrophysiological characteristics of the NPRL2 nKO neurons suggested a dysregulation of sodium ion channel expression could contribute to the increased amplitude of the AP. We used qRT-PCR to measure the expression of sodium channels in the brains of P16 NPRL2 nKO and littermate controls. We observed a significant upregulation of *Scn1A*, *Scn1B*, *Scn2A*, and *Scn2B* in the NPRL2 nKO compared with control, but no change in *Scn3A, Scn3B, Scn4B, or Scn5A* (Fig. 6*A*). Expression of potassium channels: *Kv1.1, Kv1.3, Kv1.4, and Kv2.2* were not significantly changed, whereas *Kv1.6* was induced 1.2-fold (Extended Data Fig. 6-1). We next performed Western blot analysis on membrane fractions and observed a significant increase in SCN1A protein expression in the NPRL2 nKO, compared with internal control (Fig. 6*B*).

10.1523/ENEURO.0317-21.2022.f6-1 Extended Data Figure 6-1 Regulation of potassium channel expression. ***A***, qRT-PCR analysis of potassium channels (*Kv1.1*, *Kv1.3*, *Kv1.4*, *Kv1.6*, *Kv2.2*) in control and NPRL2 nKO brain at P16 (*n* = 5/4, respectively). *b*, p < 0.01. Download Figure 6-1, EPS file.

We next hypothesized that mTORC1 activation contributes to the expression of sodium channels in neurons. We generated primary neuron cultures from *Nprl2^loxP/loxP^* animals and infected them with either Cre-expressing lentivirus or negative control virus. Neurons were treated at DIV 7 with rapamycin, an inhibitor of mTORC1 kinase activity, and qRT-PCR was used to measure the expression of *Nprl2* and *Scn1A*. Treatment of *Nprl2^loxP/loxP^* neurons with Cre-expressing lentivirus produced a >90% reduction of *Nprl2* and a significant up-regulation of *Scn1A*, compared with control infected neurons (Fig. 6*C*). Moreover, rapamycin treatment blocked the upregulation of *Scn1A* in NPRL2 knock-out neurons (Fig. 6*C*). These observations support an important role of NPRL2 and mTORC1 activity in the regulation of *Scn1A* in neurons.

## Discussion

Human mutations in NPRL2 are associated with a spectrum of neurologic disorders, including autism, epilepsy, and SUDEP. Here, we show that loss of NPRL2 in glutamatergic neurons causes early lethality following seizures, increases the strength of electrically stimulated APs, and significantly alters amino acid homeostasis in the brain. Remarkably, the onset of seizures in the NPRL2 nKO mice occurred shortly before the start of the light-cycle, consistent with observations of GATOR1-dependent nocturnal seizures in humans (Picard et al., 2014; Korenke et al., 2016). These circadian differences between human and mouse observations could be because of their diurnal versus nocturnal nature. While human genomic sequencing studies have identified a greater numbers of disease-linked mutations in the larger GATOR1 subunit called DEPDC5 (Baldassari et al., 2019) compared with NPRL2, our data show that strict loss-of-function mutations in NPRL2 cause early lethality and may be underrepresented as a genetic risk factor for SUDEP. As previous studies have showed global deletion of NPRL2 results in embryonic lethality and defective fetal liver hematopoiesis (Dutchak et al., 2015), the mechanisms that underlie epilepsy-linked NPRL2 somatic mutations may elicit distinct changes in the GATOR1 structure that impair cell-type specific mTORC1 regulatory functions. Collectively, these data support genetic screening of GATOR1 mutations during risk assessments for SUDEP.

GATOR1 functions as an evolutionarily conserved intracellular amino acid-signaling complex that has a critical function to maintain intracellular amino acid homeostasis (Bar-Peled et al., 2013; Dutchak et al., 2015; Chen et al., 2017; Dutchak et al., 2018). Previous studies have shown that defects in NPRL2 cause significant changes in cellular glutamine and glutamate homeostasis, and alter the expression of metabolic enzymes that control these reactions (Dutchak et al., 2018). Here, we show that deletion of NPRL2 from glutamatergic neurons causes a 60% reduction in brain glutamine levels, but an insignificant decrease in glutamate levels. These data suggest that the absence of NPRL2 in glutamatergic neurons, the high demand of glutamate to support neuronal functions, including synaptic transmission and protein translation, can be met by increased metabolic supply of glutamine from astrocytes (Schousboe et al., 2014) or potential rewiring of secondary metabolic pathways (Chen et al., 2017). Since the glutamate-glutamine cycle relies on glutamine from astrocytes to support neural populations and prevent neurotransmitter depletion, these data suggest that the ability of astrocytes to regenerate glutamine is insufficient when NPRL2 is impaired in neurons (Schousboe et al., 2013; Eid et al., 2016). Subsequently, these metabolic deficiencies may contribute to the changes in excitatory glutamate signaling associated with seizures (Schousboe et al., 2014; Eid et al., 2016), or by substrate deficiencies in the inhibitory GABAergic system (Swaminathan et al., 2018).

We also identified other amino acids deficiencies, including arginine, which may represent homeostatic mechanism to downregulate mTORC1 activity in the brain (Chantranupong et al., 2016). We did not detect any change in creatinine or taurine in the NPRL2 nKO, indicating these secondary pathways of energy metabolism are not affected. However, the increase in putrescine indicates a neurodegenerative metabolic process may be stimulated before death, whereas no changes in genetic markers of apoptosis were observed in living mice at P16 (Chiang et al., 2001; Takao et al., 2006). No changes in brain acylcarnitines, sphingolipids, lysophosphatidylcholines or phosphatidylcholines were observed. Collectively, these data support NPRL2 as a specific regulator of amino acids and their metabolic pathways in the brain.

Early studies showed patients with activating mutations in mTORC1 signaling presented with focal cortical dysplasia and neurodevelopmental cortical malformations (Lim et al., 2015; Mirzaa et al., 2016), with recent evidence supporting a novel two-hit mosaic mutation mechanism as the etiology of the focal cortical dysplasia (Ribierre et al., 2018). While no gross morphologic differences were apparent in the NPRL2 nKO brain, Layer IV–V cortical neurons of the NPRL2 nKO mice had larger cell bodies, with reduced dendritic branching, similar to previous reports when DEPDC5 is deleted using a pan-neuronal Cre-driver (Yuskaitis et al., 2018). These data support a critical role for strict mTORC1 regulation by the GATOR1 amino acid signaling pathway in early neuronal development.

Our data show that loss of NPRL2 in glutamatergic neurons is sufficient to increase mTORC1 signal transduction, despite in an intact TSC1/2 complex (Bourdeau Julien et al., 2018). Previous studies have shown that mTORC1 is localized to the axons and dendrites of neurons (Takei et al., 2004; Terenzio et al., 2018), but its biological impact has not been clearly defined. Our data support an important link between hyperactive mTORC1 and changes in the electrical activity of the neurons by affecting the expression of sodium channels. Our electrophysiological analysis shows that loss of NPRL2 is sufficient to increase the strength of evoked APs, with a significantly greater amplitude and faster rise time during the depolarization current step. Consistent with these changes in electrophysiological parameters, we observed a significant upregulation of *Scn1A, Scn1B, Scn2A, and Scn2B* in the brain of the NPRL2 nKO mice. Our loss-of-function studies in primary neuronal cultures also show consistent up-regulation of *Scn1A* expression when NPRL2 is deleted, consistent with a cell autonomous effect. Moreover, the upregulation of *Scn1A* expression is prevented by treatment with rapamycin, the pharmacological inhibitor of mTORC1. As genetic studies have identified >700 mutations in genes that code for voltage-gated sodium channels in epilepsy (Kaplan et al., 2016), and reports of GATOR1 and *Scn1A* mutations contributing to temporal epilepsy (Baldassari et al., 2016), our data now implicate the NPRL2 and mTORC1 regulatory pathways in controlling Scn1a expression.

Multiple mutations in several voltage-gated sodium channels have been linked to human epilepsy (Menezes et al., 2020). For example, the gain-of-function T226M mutation in Scn1a has been found in children with developmental and epileptic encephalopathy (Berecki et al., 2019) and gain-of-function mutations in Scn2a have been shown to elicit seizures, behavioral arrest, and behavioral abnormalities in mice (Kearney et al., 2001). The phenotype of our NPRL2 nKO model is consistent with the upregulation of Scn1A causing a gain-of-function effect. In contrast, loss-of-function Scn1A mutations are associated with genetic epilepsy with febrile seizures plus (GEFS+) and Dravet syndrome, consistent with decreased activity of inhibitory GABAergic neurons (Escayg and Goldin, 2010; Ruffolo et al., 2018). Future research investigating the contribution of voltage-gated sodium channels in distinct neuronal sub-types and brain regions will be of interest for targeted pharmacological therapies.

Genomic studies have shown that 8% of patients carrying DEPDC5 mutations present with depression (Baldassari et al., 2019). Our metabolite analysis of the NPRL2 nKO brain show significantly lower levels of the neurotransmitter dopamine, which is linked to depression (Dunlop and Nemeroff, 2007). Intriguingly, animal studies have shown that dopamine levels are decreased in rat models expressing epilepsy linked *Scn1A* mutations (Ohmori et al., 2014), providing a functional link between the overexpression of *Scn1A* and reduced dopamine levels in the NPRL2 nKO model. Collectively, these observations suggest a novel mechanism linking mTORC1 and the expression of sodium channels and the mesolimbic dopamine reward pathway.

In summary, we have shown that the NPRL2 expression in glutamatergic neurons is essential to prevent seizures before death at P20, and contributes to the regulation of amino acid homeostasis and sodium channel expression in the brain. While the impact of altered amino acid metabolism in the brain remains under investigation, our findings represent a novel link between cellular metabolic regulation and neuronal activity. We conclude that NPRL2 functions as an important regulator of mTORC1 activity that controls the expression of epilepsy-linked *Scn1A* expression to regulate the strength of neuronal APs.

## Synthesis

Reviewing Editor: William Stacey, University of Michigan Decisions are customarily a result of the Reviewing Editor and the peer reviewers coming together and discussing their recommendations until a consensus is reached. When revisions are invited, a fact-based synthesis statement explaining their decision and outlining what is needed to prepare a revision will be listed below. The following reviewer(s) agreed to reveal their identity: Jeffrey Calhoun, Jonathan Lipton. Note: If this manuscript was transferred from JNeurosci and a decision was made to accept the manuscript without peer review, a brief statement to this effect will instead be what is listed below.

From the reviewers’ comments, please note that the manuscript in its current form will be helped by a more cohesive narrative and adequate discussion of the concerns raised

## Author Response

Dear Dr. Ukpong Eyo:

Please find the revised manuscript of eN-TNWR-0317021X, which now includes several new data sets and details to address the reviewers comments. We believe these changes have significantly strengthened our manuscript and thank the reviewers for their comments.

In addition to textual changes, we now include new data to address the questions raised. We have used immunohistochemistry, staining neurons and glia cells to examine P-S6 induction in a cell autonomous manner, quantitative RT-PCR to measure genetic markers of neuronal apoptosis, and performed extensive video recording analysis of the NPRL2 nKO animals to show that the mice have multiple seizures prior to death. We believe these new data support a critical role of NPRL2 in SUDEP, within glutamatergic neurons.

We have also changed the title to better reflect the data that is presented in the paper. Our new title “NPRL2 Inhibition of mTORC1 Controls Sodium Channel Expression and Brain Amino Acid Homeostasis.”, omits “Sodium Channel-Dependent Action Potentials” from our previous title. To address a comment raised by the reviewer, we pursued studies to measure sodium channel currents in vitro using primary neuron cultures, in collaboration with Dr. Mohamed Chahine’s, an expert in sodium channel function at our research center. The model of NPRL2 loss of function that we tested was generated by Cre-virus, or control, infection in NPRL2-floxed primary neuron cultures, which are distinct from the in vivo model discussed in our paper. As mentioned by the reviewer, the timing of sodium-channels expression is known to be developmentally and temporally regulated; and our results also show the metabolic context in vivo differs significantly between control and NPRL2 nKO brain, adding complexity to address this question in an in vitro system. We measured the sodium current at DIV6 using two different concentrations of sodium, but did not find a statistical difference when the data was normalized to cell capacitance. We anticipate extensive studies or the generation of a new mouse model with GFP-labeled glutamatergic neurons will be required to provide a suitable model for these future studies.

We thank you for your kind receipt and consideration of this manuscript.

Sincerely,

Paul Dutchak, Ph.D.

-

REVIEWER 1

The authors present a new neuronal (glutamatergic) conditional Nprl2 knockout mouse model with carefully performed experiments to demonstrate changes in cell size, mTORC1 signaling, and intrinsic electrophysiological properties, among other properties. This study is likely to be of interest to the scientific community, especially those interested in epilepsy and mTORopathies. There are a few components missing from this study that could strengthen this manuscript. Please see concerns in bullet point below:

-The assays performed in Fig 1A and 1B are convincing to demonstrate reduced Nprl2 expression. I found it quite interesting the difference in phenotype between the global knockout previously published (pups not born) relative to this study (mice born but with lethality around the age of weaning). I may have missed it, but might be worth highlighting this difference in the manuscript.

We thank the reviewer for this insightful comment. We now include a description of the embryonic lethality and defective fetal liver hematopoiesis described for the global NPRL2 knockout mouse. We also acknowledge that tight regulation of mTORC1 signal transduction is necessary outside of the CNS, where altered mTORC1 activity has been implicated in cancer and developmental defects.

-The mice dying around weaning is suggestive of SUDEP, which is quite interesting given that NPRL2 patients are at risk for SUDEP. More thorough phenotypic characterization of the mice would be of interest. Are these mice having seizures? In particular focal seizures? Is there any evidence from video recording, EEG, or even observation of hindlimb extension in deceased pups that the mice are dying after a seizure? Probably beyond the scope of this study, but this study certainly begs the question whether treatment with sodium channel inhibitors might be particularly useful in NPRL2-related epilepsy. Without a more thorough phenotypic characterization, it is unclear whether this mouse model will be of use in testing this particular hypothesis.

The gold-standard of seizure detection is by video recording with EEG monitoring, which is a technical limitation with the age of lethality in our animals. We have now performed video recordings of the mice, with their mothers, and show new data that the mice do have seizures. The length and frequencies of the seizures are now reported, with mice succumbing from a seizure approximately 4 hours after the first seizure is detected. We agree that pharmacological approaches to impair seizures and prevent SUDEP are of interest, but are outside the scope of this current study.

-The experiments performed all point to a change in sodium channel expression in nNprl2 neurons. What is missing are sodium current recordings from slice or acutely dissociated neurons to investigate the levels of functional sodium channels in the membrane in nNprl2 neurons relative to littermate controls. Many sodium channels present in cells are inactive and not present at the plasma membrane (PMID: 2410908; doi: 10.1073/pnas.82.14.4847) and these experiments would be useful to show that the increased RNA and protein observed translates to elevated glutamatergic sodium currents.

We thank the reviewer for raising the comment regarding the biochemical observations of SCN1 subunit expression and its interactions during development. Consistent with our in situ electrophysiological recordings that show increased action potential strength in the NPRL2 nKO mice evoked by depolarization current, our biochemical data show increased SCN1 protein expression in the membrane fractions. Since our animal model is not amenable to acutely dissociated neurons (not all neurons are VGLUT2-positive and the mouse does not carry a GFP marker), we have measured sodium currents in DIV6 primary neuron cultures from the NPRL2-floxed mice that were infected with Cre expressing virus, or control virus, aiming to limit spontaneous firing artifacts of the developing circuitry. Two different extra cellular sodium concentrations (107mM and 35mM) were tested and at these two concentrations we did not observe any statistical difference when current were normalized to cell capacitance. As indicated by the reviewer, the timing of SCN expression is important developmentally; as well as the context of the metabolic differences between the cellular media (in vitro) and the brain metabolism (in vivo) highlighted in Figure 5. Determining these variables on sodium currents in vitro will be of interest for future investigations that extend beyond this study.

REVIEWER 2

In this paper, the authors investigate a mouse model of NPRL2 loss of function in a subset of central glutamatergic neurons. Because NPRL2 is part of the GATOR1 complex that acts as a negative regulator of amino-acid dependent mTOR function, the authors hypothesize that a loss of function model will uncover new insights into NPRL2 and GATOR1 function in the brain. This is of potential clinical significance because NPRL2 mutations have been associated with various forms of epilepsy.

My overall impression is that this is a well-written paper that presents a nice set of mostly descriptive data from a new model of mTOR dysfunction. The findings themselves are convincing and nicely presented but they support only a fraction of the conclusions made by the authors and are not mechanistically linked together into a convincing narrative. This limits the impact of the data.

We thank the reviewer for the comments. We hope our observations will provide new insights regarding the mechanistic and biological functions of NPRL2 and mTOR dysfunction from our novel model. We have now revised the manuscript and provided new data that we believe has significantly strengthened the work.

Specific comments on figures and related text follows.

Figure 1. The western data clearly shows a limited expression of NPRL2 but it would have been nice to see this by immnohistochemistry. Is the decreased expression limited to vGLUT2-positive cells or is it global? Without this data, it is hard to interpret many of the later findings. It is unclear why mTOR signaling was not included in this first figure as it would set up the rest of the manuscript in a much more effective way.

We agree that immunohistochemistry of NPRL2 would provide additional support to our gene expression (QPCR) and protein analysis (western blotting) of NPRL2 expression. However, no commercially available antibodies exist for validated NPRL2 IHC staining. We have now included more detailed description of the Cre-recombinase mice (The Jackson Laboratory, Stock No. 028863), including information that Cre expression is driven by an IRES that was created using a knock-in strategy into the Vglut2 gene. We also detail previous publications using the NPRL2 floxed allele, which have shown the tissue specific excision of the floxed-NPRL2 by tissue dependent Cre expression (DOI: 10.1016/j.celrep.2018.04.058). The global NPRL2 KO mouse is embryonic lethal and with defective hematopoiesis, which we now have clarified in the text (DOI: 10.1016/j.celrep.2015.06.042).

Figure 2. Does rapamycin reverse the changes in cell size or is this phenomenon static?

We thank the reviewer for raising this interesting question. The activity of mTORC1 signaling is known to control cell size, such that higher mTORC1 activity correlates with increased cell size and reduced mTORC1 activity correlates with smaller cell size. Previous reports show that rapamycin can inhibit mTORC1 signaling, independent of NPRL2 (DOI: 10.1016/j.celrep.2015.06.042), consistent with rapamycin functioning as an allosteric inhibitor of mTORC1. With recent publications showing that the timing of rapamycin treatment is critical to effect structural defects causes by mTORC1 activation (DOI: 10.3389/fnmol.2018.00409), we believe that investigation of rapamycin effects on NPRL2-deficient neuronal cell size extend beyond the scope of this manuscript.

Figure 3. Why is the physiology performed in the lateral habenula? I could not find any justification in the text for testing this particular area.

We selected the lateral habenula because the neurons in this region are exclusively VGLUT2 positive. We have now added this information in the text.

Figure 4. This figure would be better incorporated into FIgure 1. It would also be nice to see more detailed analysis of cell types in the IHC, perhaps with cell-specific co-stains. The change in pS6 is almost impossible to see by IHC. Is the change in pS6 exclusively in neurons? What about astrocytes or glia? How would the authors explain a cell autonomous vs non-autonomous effect without having this data?

We have now separated the channels of the IHC and believe the staining signal intensity is more apparent in this form. We recognize that IHC is not a quantitative method and subsequently believe that the supportive western blot analysis fits more closely to the histological analysis than the phenotypic characterization of the mice in Figure 1. We have now included new data showing the co-staining of P-S6 with NeuN and GFAP to support the cell autonomous effect.

Figure 5. Since virtually all amino acids change in the cortical metabolome, how do the authors rule out the possibility that this is not secondary to massive cell death?

We agree that the decreased abundance of many amino acids in the cortical metabolome are striking. These data, however, are consistent with previous studies of NPRL2-knockout in other tissues (doi: 10.1016/j.celrep.2018.04.058) and support our model that NPRL2 functions as a major homeostatic regulator of cellular amino acid metabolism. We now clarify that tissues were collected from live P16 animals, with no physical signs of stress or movement impairment. We also include new data regarding gene expression of markers associated with the transcriptional response of cell death.

Figure 6 and related. In the final section the authors hypothesis that the electrophysiological defect in NPRL2 nKO neurons could be explained by “dysregulation of sodium ion channel expression”. While this is certainly true, it is not clear why sodium channel disruption could be any more involved than other channels. In other words, the justification for the next set of experiments is unclear. In Fig 6B why is SCN1A the only western blot included? In fig 6C what is the explanation for the transcriptional change in Scn1A gene expression? What is the mechanism of NPRL2-mediated transcription? Is this change cell autonomous? How does the Scn1A increase correlate with electrophysiological changes? Is this somehow related to amino acid processing?

We thank the reviewer for this series of questions. The electrophysiological analysis of the NPRL2 nKO neurons show increased depolarization, which is occurs from the movement of sodium ions through sodium channels. We focused our investigation on SCN1A because of its well described links with a spectrum of seizure-related disorders in humans, with over 150 mutations linked to epilepsy. Moreover, it has been reported to contribute to temporal epilepsy like NPRL2, NPRL3 and DEPDC5 mutations from clinical genomic sequencing studies of epilepsy (doi.org/10.1136/jmedgenet-2016-103883). We have clarified the text by indicating that other channels may contribute to the electrophysiological properties of the neurons. The changes in gene expression in our primary neuron culture experiments support that the genetic regulation is occurring in a cell autonomous manner, and we have clarified this point in the discussion. As NPRL2 is not a transcription factor itself, we anticipate that transcriptional changes elicited by its loss of function are driven by signaling events that modulate particular downstream transcription factors, likely mediated through mTORC1 because treatment with rapamycin, an mTORC1 inhibitor, blocks the induction.

There are many interesting findings in this manuscript findings but the lack of mechanistic threads that connect them, or at least, clear justifications linking each step obfuscates the impact of the findings.
